# Current status of biological control of introduced *Phragmites* in Canada: Insights from initial years of post-release monitoring and a larval density release experiment

**DOI:** 10.1371/journal.pone.0315071

**Published:** 2024-12-18

**Authors:** Michael J. McTavish, Ian M. Jones, Sandy M. Smith, Robert S. Bourchier

**Affiliations:** 1 Institute of Forestry and Conservation, John H. Daniels Faculty of Architecture, Landscape and Design, University of Toronto, Toronto, ON, Canada; 2 Agriculture and Agri-Food Canada, Lethbridge, Alberta, Canada; University of Ferrara, ITALY

## Abstract

Introduced *Phragmites* (*Phragmites australis australis* (Cav.) Trin. Ex Steud.) is one of the most invasive plants in North America. To supplement existing management tools, a classical biological control program began in Canada in 2019 using two host-specific stem-boring moths, *Archanara neurica* (Hübner) and *Lenisa geminipuncta* (Haworth) (Lepidoptera: Noctuidae). In this article, we summarize the first three years of monitoring data for *L*. *geminipuncta* and *A*. *neurica* as biological control agents for introduced *Phragmites*. First, we assess agent presence and activity in the initial years post-release based on feeding damage from long-term monitoring data across 30 release sites initiated between 2019 and 2023. Second, we investigate the within-site distribution of agent feeding damage to improve future monitoring and agent collection from nurse sites. Third, we report the results of an experiment to determine optimal release densities of *A*. *neurica* larvae. We found agent feeding damage at 92% of initial release sites in the first year and agent activity persisted at all of these sites into years two and three post-release. Patterns of agent feeding damage suggest that the agents disperse quickly through the patch following release, favouring the interior area over the edges of introduced *Phragmites* stands. Finally, releasing intermediate densities of 40 *A*. *neurica* larvae per release point was more efficient than releasing either units of 20 or 80 larvae. The results of the first three years of monitoring are highly encouraging for the introduced *Phragmites* biological control program. Insights from these early monitoring results will be used to refine optimal release strategies, improve our ability to locate egg-bearing stems at nurse sites to facilitate the collection and redistribution of agents to new release locations, and inform protocols for longer-term monitoring of impacts on the target weed once agents are established.

## Introduction

Common reed (*Phragmites australis australis* (Cav.) Trin. Ex Steud.)–hereafter referred to as “introduced *Phragmites*”–is one of the most invasive plants in North America. Arriving from Eurasia in the late 19th century, the weed is now widespread along the Atlantic, Pacific, and Gulf Coastal regions [1, 2], with the potential for significant further range expansion [3]. Introduced *Phragmites* is a perennial grass that forms dense stands in wetland and riverine habitats, as well as in roadside ditches [4, 5]. These dense stands cause significant reductions in native plant diversity [6], with related impacts on communities of birds [7], fish [8], and turtles [9]. Along with a cascade of other negative ecological impacts [10–12], introduced *Phragmites* presents a major threat to the native North American subspecies of common reed (*P*. *australis americanus* Saltonstall, P.M. Peterson & Soreng) through both competition [2, 13] and hybridization [14, 15].

Conventional control methods for introduced *Phragmites* have seen limited success [16–18]. The use of herbicides is prohibited in many invaded sites due to threats to biodiversity, human health, and water quality [19–22], while physical control methods such as cutting and burning are highly labour-intensive and are only practical at relatively small scales. There is a clear need for additional management options for introduced *Phragmites*. Classical biological control represents a scalable and sustainable tool to be used as part of an integrated management approach [23].

The biological control program for introduced *Phragmites* began in 1998 with assessments of the weed as an appropriate target and a search for candidate agents [24–26]. Two European stem-boring moths, *Archanara neurica* (Hübner) and *Lenisa geminipuncta* (formerly *Archanara geminipuncta*) (Haworth) (Lepidoptera: Noctuidae), were identified as promising candidates by the Centre for Agriculture and Biosciences International (CABI) in Delémont, Switzerland. Host-range testing conducted between 2005 and 2018 demonstrated that both species are specific to introduced *Phragmites* [27, 28]. *Archanara neurica* and *L*. *geminipuncta* share similar life histories with a few key differences in phenology and feeding behaviour. Both species are univoltine, overwintering as eggs laid under the leaf sheaths of introduced *Phragmites* [29, 30]. Larvae emerge in the spring and enter a young stem in which they feed on the tissue above the growing meristem [31]. Larvae typically feed on three shoots as they move through four instars (*A*. *neurica*) or four shoots through five instars (*L*. *geminipuncta*), inflicting varying degrees of damage on each host stem [29, 32]. Young stems attacked by early instars generally die, while older stems attacked by later instars suffer wilted stem tips and rarely produce flowers [32, 33]. During early instars, as many as ten *L*. *geminipuncta* larvae can be found feeding in a single stem, while *A*. *neurica* are strictly solitary feeders [32]. Mature larvae pupate in a new shoot and adults emerge after approximately 26 days (*A*. *neurica*) or 39 days (*L*. *geminipuncta*) [32]. *Lenisa geminipuncta* is the most common pest of *Phragmites australis* in Europe and has been observed to damage up to 90% of stems leading to reductions in above-ground biomass of 20 to 60% [34].

A petition to release both agents in Canada and the USA was submitted in 2018 [35]. In the USA, the insects were recommended for release by the USDA-APHIS Technical Advisory Group and are currently at a subsequent review step in the US regulatory process. In Canada, the Canadian Food Inspection Agency issued a release letter in 2019 and the first field releases were conducted in Ontario, Canada the same year. Since 2019, operational release protocols have been developed for eggs and larvae, prioritizing efficient release techniques that minimize the loss of agents to predation and buffer against phenological mismatches between the agents and the host weed [36]. Using these release methods, a total of 23,400 insects have been released across 30 sites in Ontario from 2019 to 2023.

In this article, we summarize the results of the first three years of *L*. *geminipuncta* and *A*. *neurica* releases in Canada, presenting the data in three main sections. First, we provide an overview of long-term monitoring data of agent feeding damage across the 30 release sites initiated between 2019 and 2023. These monitoring data can be used to assess initial agent release success, agent persistence over multiple years through overwintering and reproduction, changes in agent activity over time, and eventual long-term establishment. Second, we investigate the within-site distribution of agent feeding damage during the initial years post-release. We used the monitoring data to compare feeding damage intensity and distribution within sites and to compare agent feeding between introduced *Phragmites* edge and interior habitats. Understanding these within-site patterns of agent feeding damage will help improve monitoring protocols and facilitate the collection and redistribution of agents from established nurse sites. Third, we report the results of a larval release density experiment conducted across eight sites in 2023. The experiment sought to identify optimal release densities that would efficiently use limited biological control agents to maximize damage to the weed while minimizing agent loss through presumed intraspecific competition. Because this biological control program is in the early stages, the availability of the two agents for releases varied greatly year-to-year. Given this and the very similar life histories of *A*. *neurica* and *L*. *geminipuncta*, we have combined monitoring data from all sites for both agents for the purposes of this study.

## Materials and methods

### Release methods and sites in Ontario

**Fig 1:**
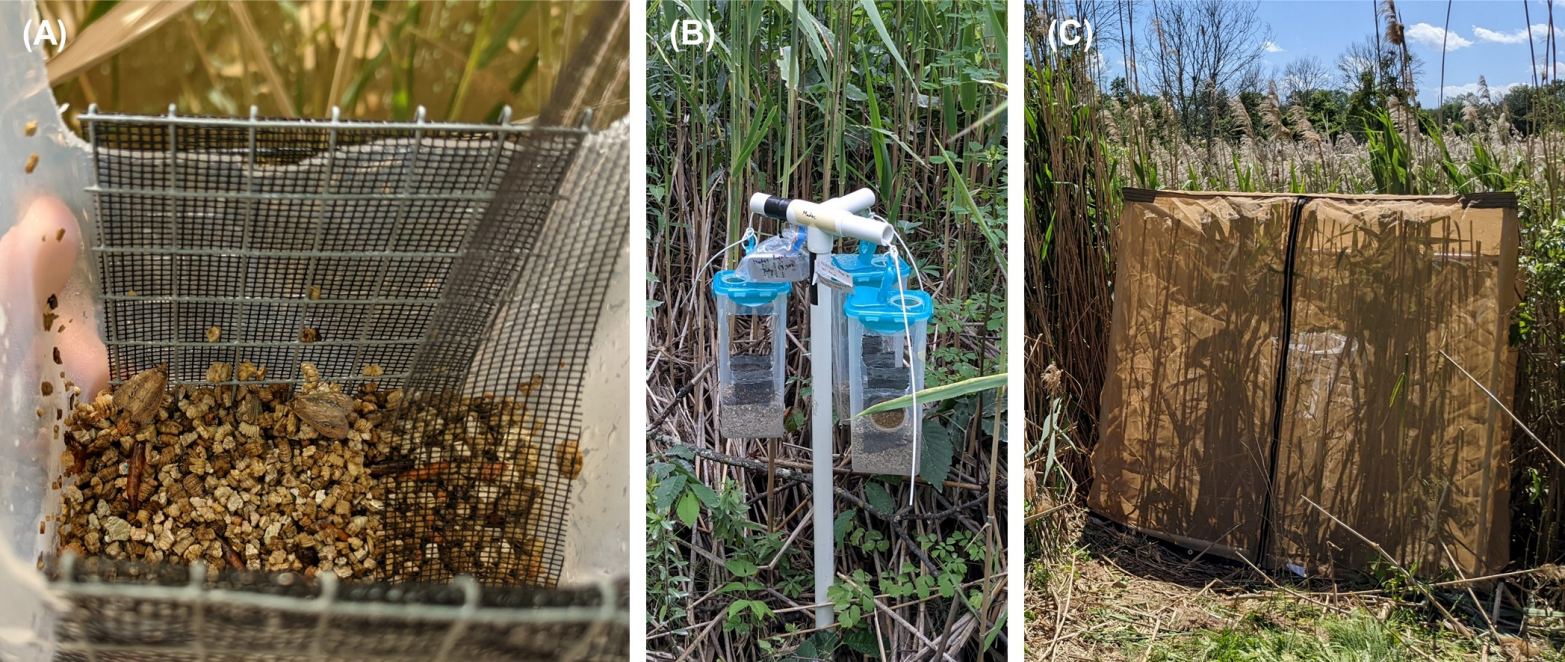
Additional release methods for *Archanara neurica* and *Lenisa geminipuncta* pupae and adults. Pupae can be placed in vermiculite in the bottom of containers with open side panels and fiberglass/metal mesh surfaces to allow emerging moths to climb up to exit (A) and then hung out in the open (B) or in mesh cages (C). Adults can be transported to the field and released directly into mesh cages (C).

From 2019 to 2023, *A*. *neurica* and *L*. *geminipuncta* have been released into the field using seven release methods spanning all life stages (i.e., eggs, larvae, pupae, and adults). Descriptions of the basic release techniques and the number of times that they have been used to date are provided (Table 1). Egg and larval release methods are described in greater detail elsewhere [36], with egg cups and stem larvae currently recommended as the best and most frequently used release techniques. Relatively few releases have been conducted with *A*. *neurica* or *L*. *geminipuncta* pupae or adults due to the additional rearing and field logistics required (Fig 1).

**Table 1:** Overview of methods used for releasing *Archanara neurica* and *Lenisa geminipuncta* from 2019 to 2023 in Ontario, Canada.

Release method	General description	Number of releases
Stem larvae	Hatching larvae are inoculated into cut *Phragmites* stems and transferred into the field in spring (~May to early June).	24
Egg cups	Eggs are placed in a closed cup with a screened bottom to exclude predators and staked in the field (in December or April to May).	10
Open pupae	Pupae are placed in open air vermiculite-filled containers and transferred to the field in summer (~June).	4
Caged pupae	Pupae are placed in open air vermiculite-filled containers and transferred to the field in mesh-enclosed cages in summer (~June).	2
Egg cards	Eggs are glued to paper cards secured to dead standing stems in the field in early spring (~April to early May).	2
Loose larvae	Larvae are scattered directly into the field amongst emerging stems within 24 h of hatching in spring (~May).	1
Caged moths	Moths are placed in portable mesh cages and transferred to larger mesh cage enclosures in the field in summer (~June).	1

Release methods are organized by frequency of use. Number of releases indicates how many times each method has been used to date at a release site.

As of fall 2023, 8,317 *A*. *neurica* and 12,401 *L*. *geminipuncta* eggs, larvae, pupae, and adults have been released using these methods at 30 locations across southern Ontario, Canada (Table 2). Site permits were obtained as required (e.g., for lands managed by conservation authorities) but were not needed for all sites (e.g., private lands, public roadsides) (S1 Table). Release sites are numerically labelled in approximate order of release date. Releases ranged from small, single plot releases designed solely to establish the biocontrol agents on the landscape, to large-scale manipulative experiments testing various operational and biological questions of interest. In most cases, a single set of releases was performed concurrently at each site, with the exceptions of several early release sites (P02, P03, P04, P05, P06) that received a second year of releases. Both species were initially released in 2020 at P02 and P03, but no eggs hatched during the 2020 season (see [36] for additional details) so agents were re-released in 2021. Because no hatch was ever observed in 2020, releases in that year were not counted towards the total numbers released. Agents were also released in 2021 at sites P02, P04, and P05, and again in 2022 for smaller experimental tests of the release methods. However, because the majority of agents at these sites were released in 2021, this was considered the baseline release year for monitoring purposes. Finally, because no activity was observed following the open pupal release at P06 in 2021, agents were re-released in 2023 and this was considered the baseline year for monitoring.

**Table 2:** Summary of *Archanara neurica* and *Lenisa geminipuncta* biological control releases from 2019 to 2023 in Ontario, Canada.

Site	Release year(s)	Release method(s)	*Archanara neurica*	*Lenisa geminipuncta*	Additional analyses
P01: Davern	2019	Caged pupae	42	-	-
P02: Aurora	2020, 2021*, 2022	Egg cards, egg cups, loose larvae, stem larvae	-	3285	Y1-2, Y2-3, DD (Y2, Y3), EI (Y3)
P03: Wainfleet	2020, 2021*	Egg cards, egg cups, open pupae	-	52	Y2-3, DD (Y2, Y3), EI (Y3)
P04: Sinclair Campbell	2021*, 2022	Egg cups, stem larvae, caged pupae	68	1995	Y1-2, Y2-3, DD (Y2, Y3)
P05: Oshawa	2021*, 2022	Egg cups, stem larvae	-	2538	Y1-2, Y2-3, DD (Y2, Y3), EI (Y3)
P06: Koffler	2021, 2023*	Stem larvae, open pupae	420	52	LD
P07: Aultsville	2021	Open pupae	-	52	Y2-3, DD (Y2, Y3)
P08: Madoc	2021	Open pupae	-	52	Y2-3, DD (Y3)
P09: Scarborough	2022	Egg cups, stem larvae	-	1275	Y1-2, DD (Y2), EI (Y2)
P10: Zoo	2022	Egg cups, stem larvae	450	-	Y1-2, DD (Y2), EI (Y2)
P11: Waterloo	2022	Egg cups, stem larvae	1450	1450	Y1-2, DD (Y2), EI (Y2)
P12: rare	2022	Egg cups, stem larvae	-	1650	Y1-2, DD (Y2), EI (Y2)
P13: Dunnville	2022	Caged moths	180	-	-
P14: Cranberry	2023	Egg cups, stem larvae	1900	-	-
P15: Mac Coutts	2023	Egg cups	780	-	-
P16: Collavino	2023	Stem larvae	840	-	LD
P17: Cooper	2023	Stem larvae	420	-	LD
P18: Whitby	2023	Stem larvae	140	-	LD
P19: Brickworks	2023	Stem larvae	280	-	LD
P20: St. Lukes	2023	Stem larvae	560	-	LD
P21: Brimblecombe	2023	Stem larvae	280	-	LD
P22: North Bay	2023	Stem larvae	149	-	LD
P23: Garrard	2023	Stem larvae	40	-	-
P24: Nichol	2023	Stem larvae	40	-	-
P25: Victoria	2023	Stem larvae	40	-	-
P26: Gordon	2023	Stem larvae	40	-	-
P27: Lakeridge	2023	Stem larvae	40	-	-
P28: Cochrane	2023	Stem larvae	40	-	-
P29: Brooklin	2023	Stem larvae	40	-	-
P30: Donkey	2023	Stem larvae	78	-	-

In sites with multiple release years, “*” indicates the initial baseline year that was used for monitoring feeding damage by the agents. Totals include the overall number of eggs / larvae / pupae / adults released for each species. The following codes indicate which sites were included in additional analyses beyond basic descriptive statistics, including the year(s) of monitoring (Y), if relevant: Y1-2 (comparison of feeding damage from year 1 to year 2); Y2-3 (comparison of feeding damage from year 2 to year 3); DD (damage distribution analyses, including comparison of damage density and coverage, and VMR); EI (comparison of feeding damage and coverage between patch edge and interior); and LD (larval density release trial).

### Overview of release site monitoring

The primary goal of this study was to assess the presence and relative activity level of biological control agents through feeding damage as a means of evaluating release success, agent persistence, and eventual establishment at release sites. The program is also developing additional monitoring protocols to track longer-term agent demographics, dispersal, impacts on introduced *Phragmites*, and plant community responses that are beyond the scope of this study. We determined that the most practical and informative primary indicator of overall agent activity to monitor across all sites was summer feeding damage by *A*. *neurica* and *L*. *gemininpuncta*. Larval stem-mining by both species produces dead or wilted stems with one or two bore holes [31]. Stem damage is most obvious in June to early July when the brown wilted stems stand out against the young green introduced *Phragmites* stems (personal observation). As a result, larval feeding damage provides an informative measure of overall biological control agent presence and activity, and it can be measured non-destructively with high confidence and low disturbance to the *Phragmites* stand and the developing biological control agents. As larvae typically remain within ~ 3 m from the point of hatching whereas moths may disperse to find new oviposition sites throughout and between patches of introduced *Phragmites* [31], we developed different monitoring protocols for assessing agent feeding damage in year one and in subsequent years; year one focuses on feeding damage immediately around release points, while monitoring in year two onwards focuses on an overall assessment of feeding damage across an entire patch of introduced *Phragmites*. Both protocols produce the same unit of measurement (i.e., number of damaged stems per m^2^) but represent differ areas of coverage (i.e., year one is only the area immediately around release points, year two is the whole patch). For both the plot and patch-level monitoring protocols, we decided to express feeding damage as a function of areas rather than as an attack rate (i.e., % of stems with feeding damage). Counts of damaged stems provide an easy to measure and interpret assessment of agent presence and activity, while stem attack rates can vary based on underlying stem density and require additional measurements that are not practically scalable to this particular patch-level monitoring protocol. However, stem attack rates may be an important component of future monitoring protocols designed to measure agent impacts on the target weed.

**Fig 2:**
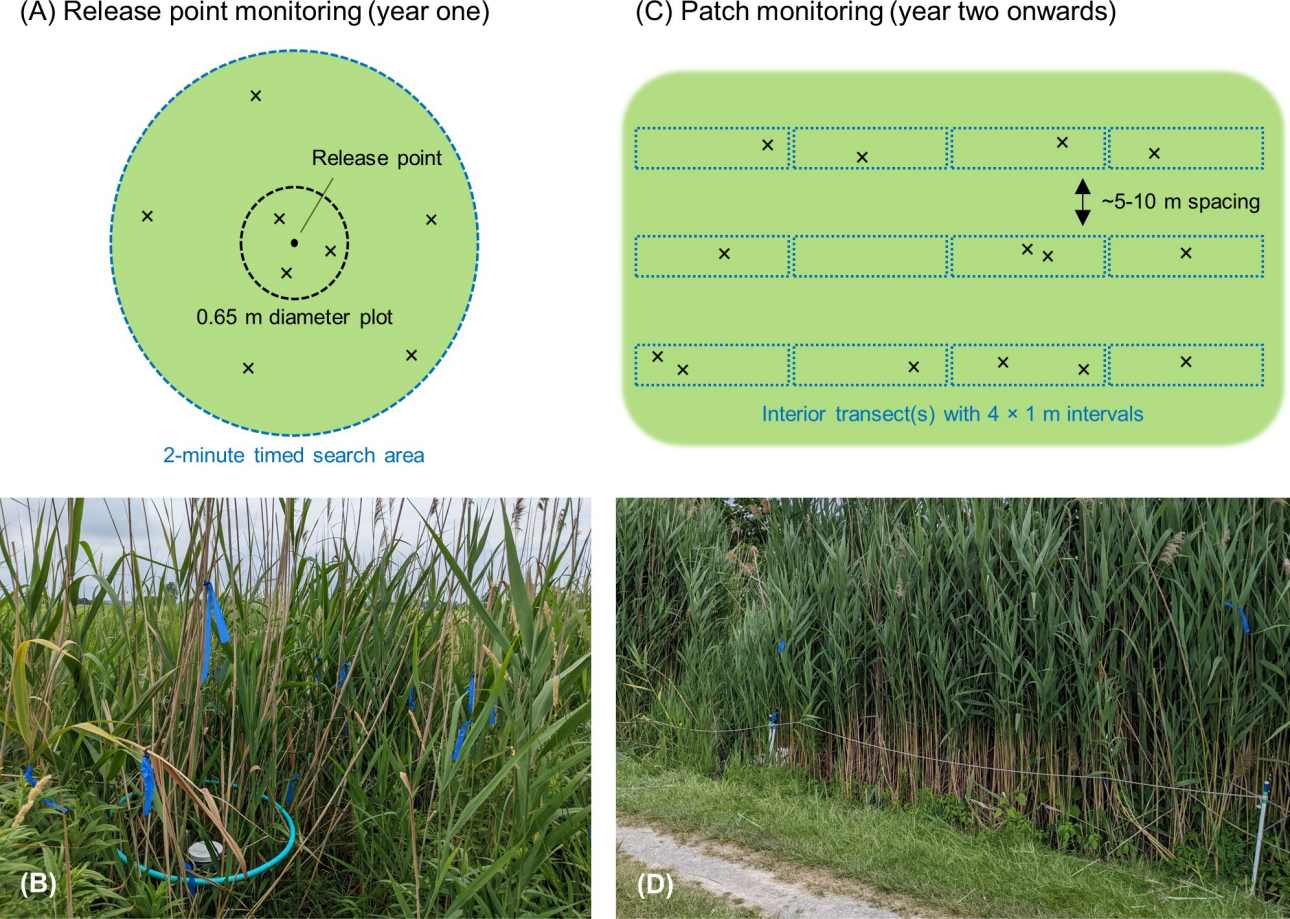
Release point and patch level monitoring protocols to assess feeding damage by released biocontrol agents. Schematic and photographic representations of monitoring protocols for (A, B) release point monitoring of new egg or larval releases in year one, and (C, D) patch monitoring of damage in subsequent years. The green background area depicts introduced *Phragmites* and “×” denotes hypothetical agent-damaged stems that would be documented by the monitoring). The number, arrangement, and length of transects were determined on a site-by-site basis to provide practical and representative coverage based on the size and shape of the introduced *Phragmites* patch.

Initial release point monitoring was used for the first year in which eggs or larvae were released at a site to determine that amount of larval feeding damage immediately around each release point. Initial feeding damage could not be assessed for releases using pupae or moths. A 0.65 m-diameter plot was established around each release point and a count was made of the number of damaged stems per m^2^. Beginning in 2022, we determined that initial release point monitoring could be enhanced by measuring the total count of damaged stems in the 0.65 m plot and found during a 2-min timed search in a 3-m radius area around the plots (Fig 2A and 2B). While we recommend continued use of this combined plot and timed search damage estimate for initial release point monitoring going forward, because timed searches were not used for release sites from 2019 to 2021, for this analysis only we report only the number of damaged stems per m^2^ from the 0.65 m plots as initial release point monitoring data.

Patch monitoring was used in subsequent years after an adult flight period had occurred and agents had a chance to disperse further within a patch of introduced *Phragmites*. This protocol was intended as an overall assessment of agent feeding throughout a patch. Agent-damaged stems were quantified in 4-m^2^ intervals (4 × 1 m) run contiguously along one or more transects spaced across the patch (Fig 2C and 2D). The number, arrangement, and length of transects were determined on a site-by-site basis to provide practical and representative coverage based on the size and shape of the introduced *Phragmites* patch. To the extent that was practical in the field, transects were run parallel with ~5- to 10-m spacing between them. Once determined for a site, transect configuration was maintained in subsequent monitoring years for within-site comparability. Of the sites that received patch level monitoring between 2022 and 2023, sites were documented using a range of 1 to 6 transects and 14 to 66 4-m^2^ monitoring intervals per site (S2 Table). Counts of damaged stems from the 4-m^2^ intervals were used to calculate mean feeding damage density (number of damaged stems per m^2^) at the site level, and damage coverage (% of 4-m^2^ intervals with one or more damaged stems).

As of 2023, the release point monitoring protocol was used at all sites where eggs or larvae were released. Patch-level monitoring was conducted at all 30 sites, annually, beginning one-year post-release. In total, release point monitoring in year one and patch monitoring in years two and three were collected from 2019 to 2023 for 25, 11, and 7 sites respectively (the changing numbers reflect the availability of sites in each category from newest to oldest and the necessary omission of releases using pupae or adults from the year one release point monitoring). These monitoring data were used to generate basic descriptive statistics characterizing agent feeding damage from the first several years of evaluation of releases. We also assessed whether feeding damage observed in a given year was a reliable predictor of damage in the subsequent year. To do this, we tested for an association between mean site-level release point year one feeding damage (number of damaged stems per m^2^ around the release point) with mean patch level year two feeding damage (number of damaged stems per m^2^ from 4 × 1 m monitoring intervals) (n = 7 sites). We then similarly tested for an association between mean patch level damage between years two and three (n = 6 sites).

### Feeding damage distribution within sites

To determine the relationship between feeding damage intensity and how it was distributed across a site, we tested for an association between site-level mean damage density and damage coverage from patch level monitoring data pooled from years two (n = 9 sites) and three (n = 6 sites). For those same 15 data collections, we also calculated average variance to mean ratio (VMR or Fisher’s Index of Aggregation) as a simple measure of spatial aggregation of feeding damage density across the 4 × 1 m monitoring intervals at each site. VMR < 1 suggests a uniform distribution, VMR = 1 suggests a random distribution, and VMR > 1 suggests aggregation.

We also used patch level monitoring data from sites in 2023 to determine whether *A*. *neurica/L*. *geminipuncta* feeding damage was more common in the interior or edge habitats of introduced *Phragmites* at the release sites. Of the sites that were monitored for patch-level feeding damage in 2023, we selected seven with larger introduced *Phragmites* patches that could be used to clearly delineate monitoring intervals placed either along the perimeter of the patch (i.e., edge habitat) or into the middle of the patch a minimum of 5 m away from the edge (i.e., interior habitat). Four were in year two of monitoring (P09, P10, P11, P12) and three were in year three (P02, P03, P05). Sites consisting of long, narrow corridors of introduced *Phragmites* were excluded because their configuration did not create a clear difference patch edges and interiors. Additional perimeter transects were placed around the introduced *Phragmites* patches at these sites as needed and monitored using the same patch-level protocol (see S2 Table for an overview of the interior and edge transects and number of monitoring intervals per site). Mean density of feeding damage and damage coverage were compared between the edge and interior transects of each site. We also tested whether the damage density or damage coverage at the edge of an introduced *Phragmites* patch could predict the amount of damage density or coverage in the interior.

### Effects of larval density on initial release success

An experiment was conducted to determine the effectiveness of releasing different densities of larval-inoculated stems (20, 40, or 80 *A*. *neurica* larvae per release). Forty stems were chosen as a practical release density previously used for releases in 2022, and 20 and 80 stems were selected as release rates half and twice as high respectively. Blocks of larvae-inoculated stems were prepared using standard methods [36]. Blocks with 20 or 40 larvae-inoculated stems or two blocks of 40 stems for 80 treatments were released across eight sites from 9 to 25 May 2023. Each site received one to six replicates of each density treatment based on the size of the site (P06 × 3 replicates, P16 × 6, P17 × 3, P18 × 1, P19 × 2, P20 × 4, P21 × 2, and P22 × 1), resulting in n = 22 replicates for the three density treatments and 66 releases in total. Stem blocks were placed ~1–5 m into the edge of each patch and separated at least 5 m between plots. At the time of the release, the density of living stems and the mean height of living stems in a circular quadrat around the release point (0.65 m diameter) were measured. Plots were monitored for initial feeding damage between June 27 and July 11, 2023 using the standard release point protocol.

### Statistical analyses

To analyze the overall site-level monitoring data, mean feeding damage from release point and patch level monitoring were calculated for all sites and summary statistics of feeding density were calculated for all three years of monitoring data. We used linear models to assess relationships between release point damage in year one and patch-level damage in year two, and between patch-level damage in years two and three. For all statistical models, unless otherwise noted, assumptions of data or residual normality and heteroscedasticity were assessed by visual inspection of quantile-quantile plots from *ggpubr* [37] and boxplots respectively. Different transformations were used on the predictor and response variables and the best fitted models were selected using AIC.

To assess within-site patterns of agent feeding damage, we created linear mixed models with *lme4* [38] to characterize the relationship between patch-level damage density and damage coverage, with follow-up monitoring year as an additional fixed effect and site as a random effect. We also used paired t-tests to compare damage density and damage coverage between edge and interior transects across the seven sites. Linear regression was then applied to model the relationship between edge/interior damage and coverage. Different transformations were used on the predictor variables and the best-fitted models were selected using AIC.

For the larval release density experiment, we assessed the effects of larval release density on total damage using linear mixed models with the *lme4* package [38]. Larval release density and the density and height of living *Phragmites* stems at the time of release were included as fixed factors and site as a random factor. Early versions of the models included interactions between all fixed effects, but these terms were not significant and were subsequently removed (data not shown). Pairwise post-hoc comparisons between larval density treatments were performed on estimated marginal means of significant models with a Tukey adjustment using *emmeans* [39]. Spearman’s correlation was assessed between living stem density and total damage to account for lack of bimodal normality using *agricolae* [40].

Unless otherwise stated, all statistical analyses were conducted using R [41] and R Studio [42] at α = 0.05. Data can be accessed online through the Zenodo repository [43].

## Results

### Overview of release site monitoring

The results of site-level release point and patch-level monitoring of *A*. *neurica* and *L*. *geminipuncta* feeding damage are presented by site (Table 3) and as summary statistics by monitoring year (Table 4). Detection of damage caused by agent feeding was high across all monitoring periods, with damage detected at all but four locations as of the most recent monitoring period in 2023. Sites with no current evidence of activity by the biological control agents include one site that received an experimental caged pupal release (P01), a municipal roadside site that was mown and destroyed post-release (P25), and two municipal roadside sites that experienced partial disturbance from mowing and had received releases of a small number of older larvae (P27, P29). Mean site-level feeding damage remained similar or increased across most sites from year two to three. Feeding damage was also initially undetected at one release site that received an open pupal release (P08) but subsequently appeared at low levels (0.1 ± 0.1 stems per m^2^) the following year.

**Table 3:** Release-point and patch-level monitoring of feeding damage caused by biological control agents released across 30 sites in Ontario, Canada between 2019 and 2023.

Site	Year 1 [release point]	Year 2 [patch level]	Year 3 [patch level]
P01: Davern	N/A	0.0 ± 0.0 (n = 14)	0.0 ± 0.0 (n = 14)
P02: Aurora	12.3 ± 16.0 (n = 11)	2.0 ± 2.4 (n = 38)	3.4 ± 2.0 (n = 39)
P03: Wainfleet	N/A	0.1 ± 0.2 (n = 18)	0.1 ± 0.2 (n = 18)
P04: Sinclair Campbell	18.5 ± 16.8 (n = 14)	5.2 ± 4.5 (n = 20)	4.7 ± 3.5 (n = 16)
P05: Oshawa	3.7 ± 8.2 (n = 9)	0.1 ± 0.3 (n = 53)	0.8 ± 1.0 (n = 42)
P07: Aultsville	N/A	0.1 ± 0.2 (n = 15)	0.5 ± 0.8 (n = 16)
P08: Madoc	N/A	0.0 ± 0.0 (n = 17)	0.1 ± 0.1 (n = 16)
P09: Scarborough	1.4 ± 3.9 (n = 11)	0.2 ± 0.3 (n = 20)	-
P10: Zoo	8.7 ± 15 (n = 3)	0.3 ± 0.4 (n = 14)	-
P11: Waterloo	14.1 ± 12.4 (n = 38)	3.3 ± 2.2 (n = 66)	-
P12: rare	12.9 ± 14.2 (n = 11)	1.8 ± 2.6 (n = 55)	-
P06: Koffler	28.1 ± 12.4 (n = 9)	-	-
P13: Dunnville	0.2 ± 0.2 (n = 6)	-	-
P14: Cranberry	11.0 ± 10.3 (n = 30)	-	-
P15: Mac Coutts	13.4 ± 8.2 (n = 9)	-	-
P16: Collavino	33.5 ± 20.3 (n = 18)	-	-
P17: Cooper	25.4 ± 13.1 (n = 9)	-	-
P18: Whitby	9.0 ± 8.0 (n = 3)	-	-
P19: Brickworks	15.6 ± 10.3 (n = 6)	-	-
P20: St. Lukes	26.1 ± 12.6 (n = 12)	-	-
P21: Brimblecombe	15.1 ± 12.9 (n = 6)	-	-
P22: North Bay	6.0 ± 10.4 (n = 3)	-	-
P23: Garrard	6.0 (n = 1)	-	-
P24: Nichol	6.0 (n = 1)	-	-
P25: Victoria	(Site destroyed)	-	-
P26: Gordon	9.0 (n = 1)	-	-
P27: Lakeridge	0.0 (n = 1)	-	-
P28: Cochrane	3.0 (n = 1)	-	-
P29: Brooklin	0.0 (n = 1)	-	-
P30: Donkey	1.5 ± 2.1 (n = 2)	-	-

Feeding damage (number of damaged stems per m^2^) is presented as mean ± standard deviation with the number of release point plots (year one) or monitoring intervals (years two and three) monitored for each site. “N/A” indicates sites for which release point larval feeding damage could not be monitored during the release year because pupae or moths were released. Dashes (“-”) indicate sites that were not old enough for data to have been collected.

**Table 4:** Summary of monitoring of feeding damage by biological control agents by year.

Monitoring period	Number of sites	Sites with damage (%)	Mean damage ± SD (stems per m^2^)	Mean damage coverage ± SD (% of intervals with damaged stems)
Year 1	25	92	11.2 ± 9.3	N/A
Year 2	11	82	1.2 ± 1.7	50 ± 38
Year 3	7	86	1.4 ± 1.9	51 ± 40

Note that year one damage is measured at the release point scale while year two onwards is measured at patch scale. Damage coverage cannot be measured during year one monitoring.

**Fig 3:**
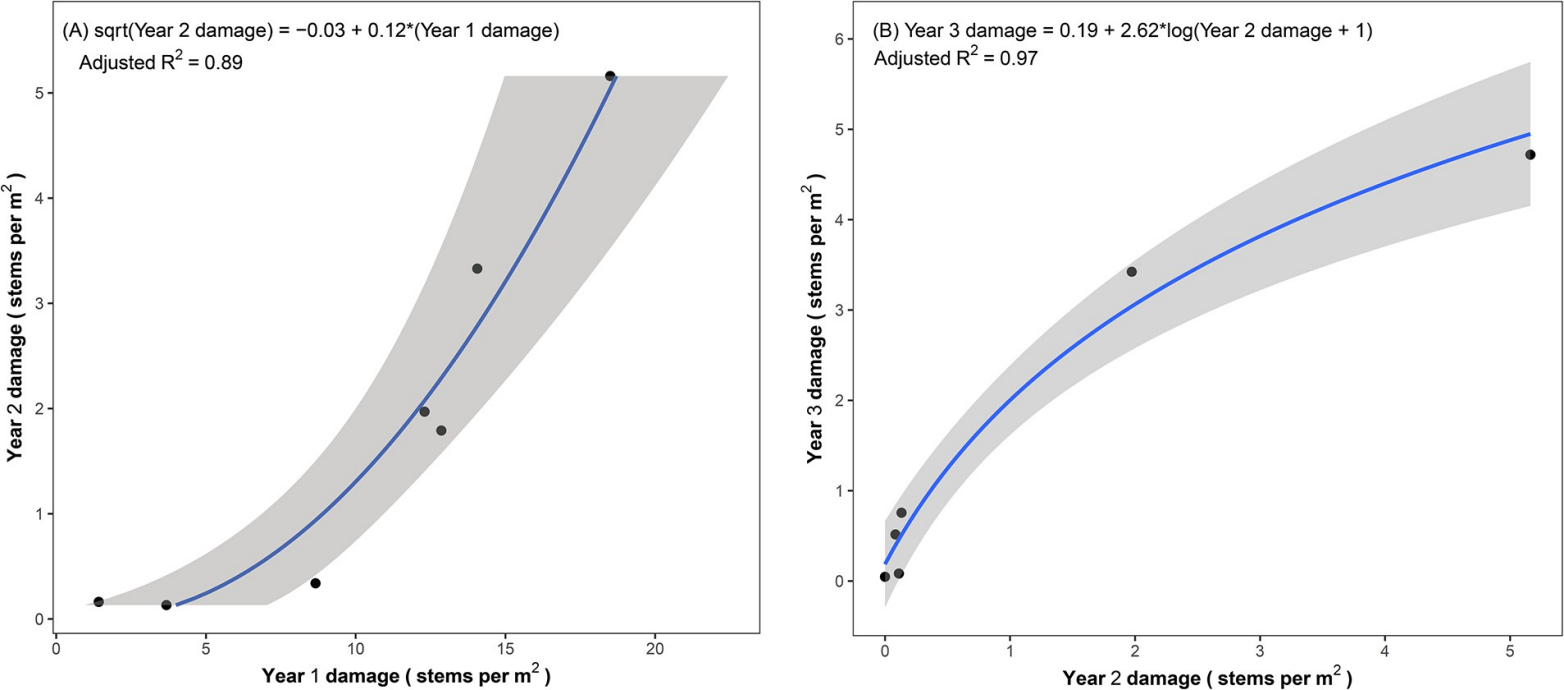
Feeding damage caused by the biological control agents in a given year predicted feeding damage in subsequent years. Scatterplots with regression lines depicting the relationships of the amount of agent feeding damage (number of damaged stems per m^2^) observed between (a) release point monitoring in year one and patch level monitoring in year two (n = 7 release sites), and (b) between the second and third years of patch level monitoring (n = 6 release sites). The shaded area depicts a 95% confidence interval.

Initial feeding damage observed during the release year was a strong predictor of the amount of feeding damage observed in year two (F_1,5_ = 47.62, p < 0.001, adjusted R^2^ = 0.89) (Fig 3A), which in turn was a strong predictor of the amount of feeding damage observed in the third year (F_1,4_ = 176, p < 0.001, adjusted R^2^ = 0.97) (Fig 3B).

### Feeding damage distribution within sites

**Fig 4:**
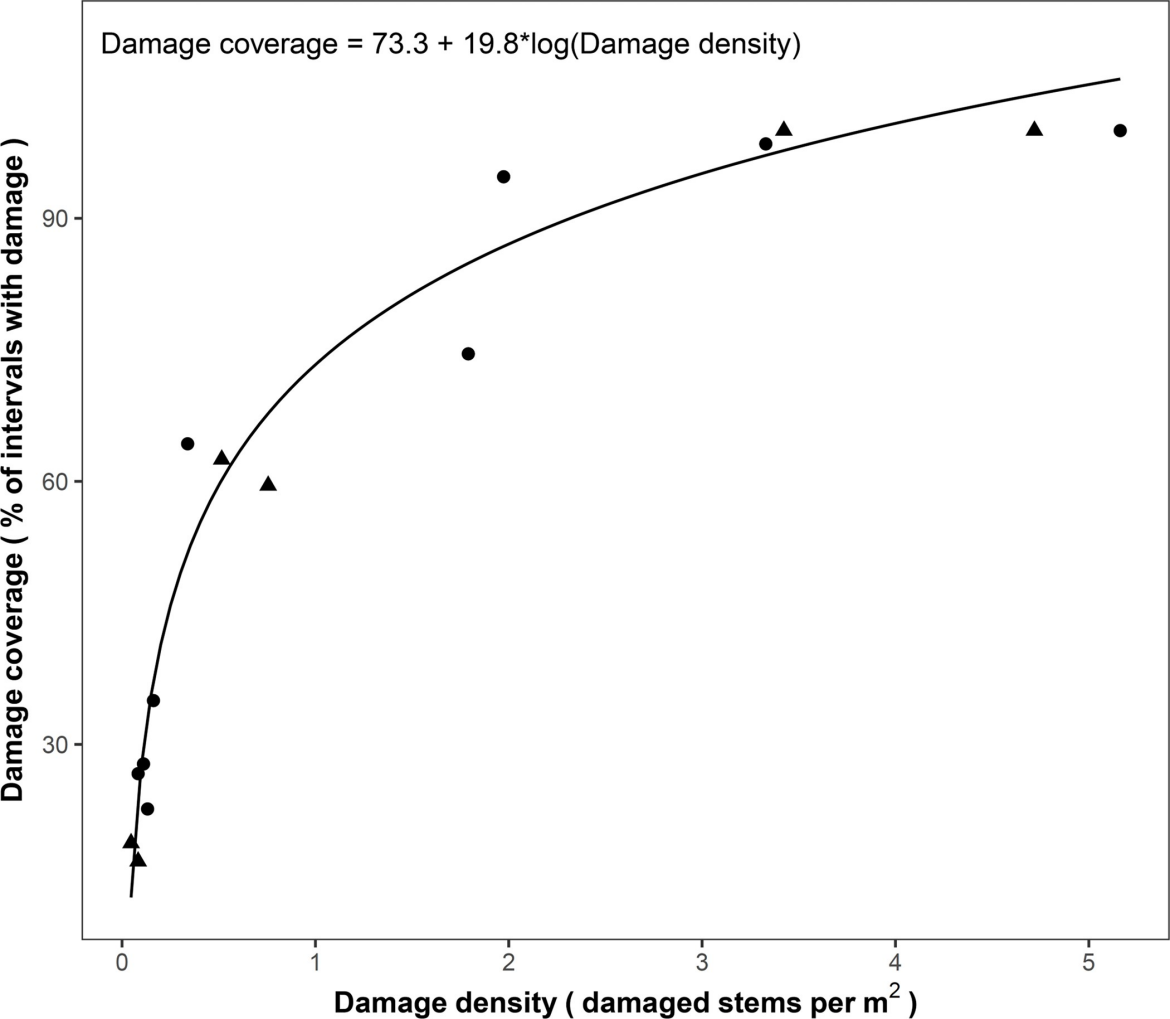
Density of feeding damage and damage coverage caused by the biological control agents was related. Scatterplot of the density of agent feeding damage (number of damaged stems per m^2^) and coverage (% of monitoring intervals with ≥ 1 damaged stems) during follow-up monitoring (total n = 15 sites, circles: year two, triangles: year three). The regression line was produced from a linear mixed-effects model of damage coverage as a function of the natural logarithm of damage density with monitoring year as a fixed effect (non-significant) and site as a random effect.

Analyzing 15 data points of patch-level monitoring from years two and three (Table 2), the damage density was a significant predictor of damage coverage (F_1,12.02_ = 332.04, p < 0.001) and damage coverage increased linearly with the natural logarithm of damage density (Fig 4). The relationship between damage density and coverage did not differ between monitoring years two and three (F_1,5.13_ = 0.47, p = 0.52). Across the same 15 data collections, mean VMR was 1.4 ± 1.2 (± SD) (range 0.22 to 3.99) suggesting stem damage was clumped or aggregated within sites.

**Fig 5:**
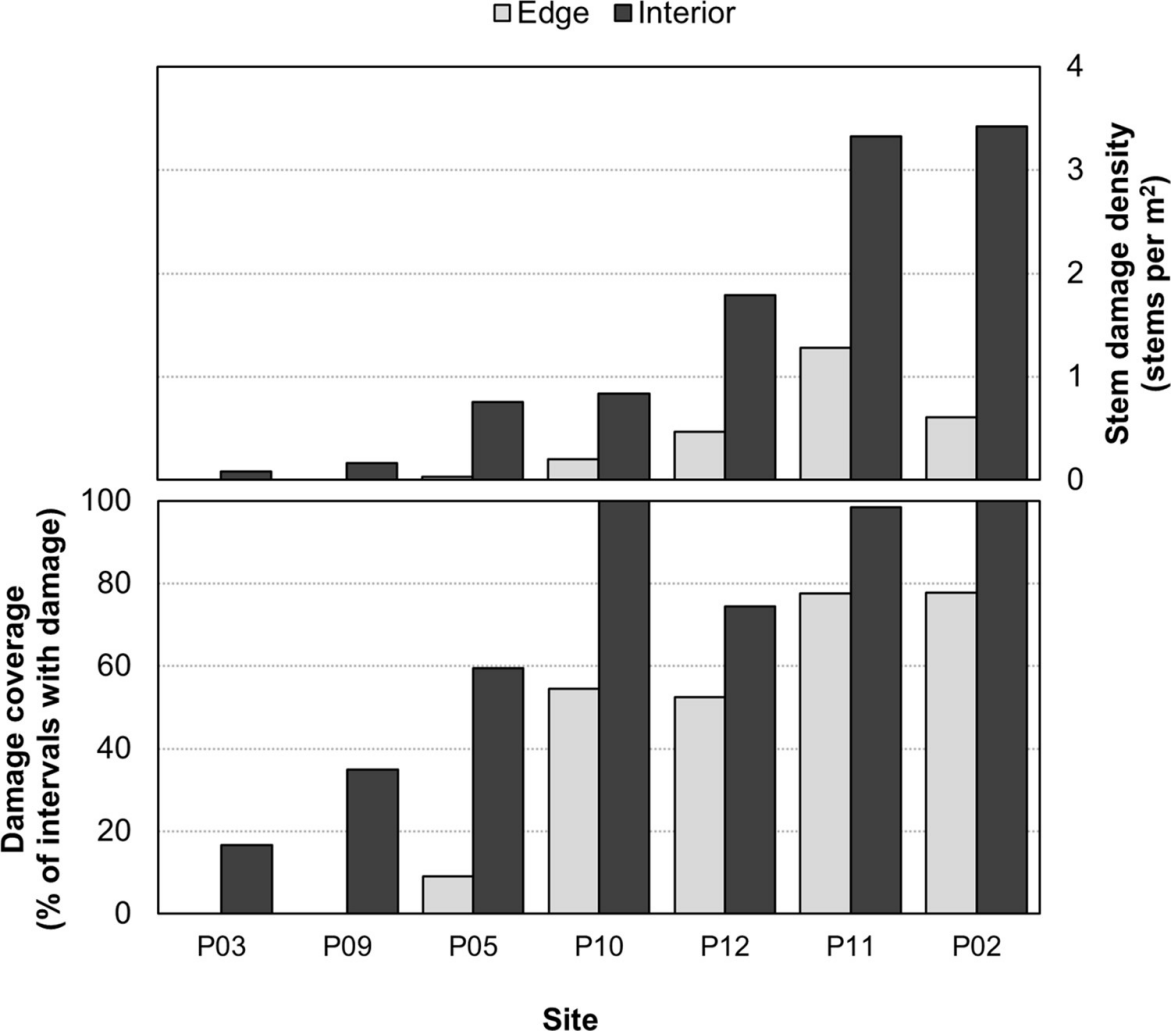
Density of feeding damage and damage coverage caused by released biological control agents were higher in patch interiors compared to edges of *Phragmites* plots. Comparison of (A) mean stem damage density (number of damaged stems per m^2^ of monitoring transect) and (B) stem damage coverage (% of 4 × 1 m monitoring transects with ≥ 1 damaged stems) between edge (light grey) and interior (dark grey) habitats at seven biological control release sites for introduced *Phragmites*.

**Fig 6:**
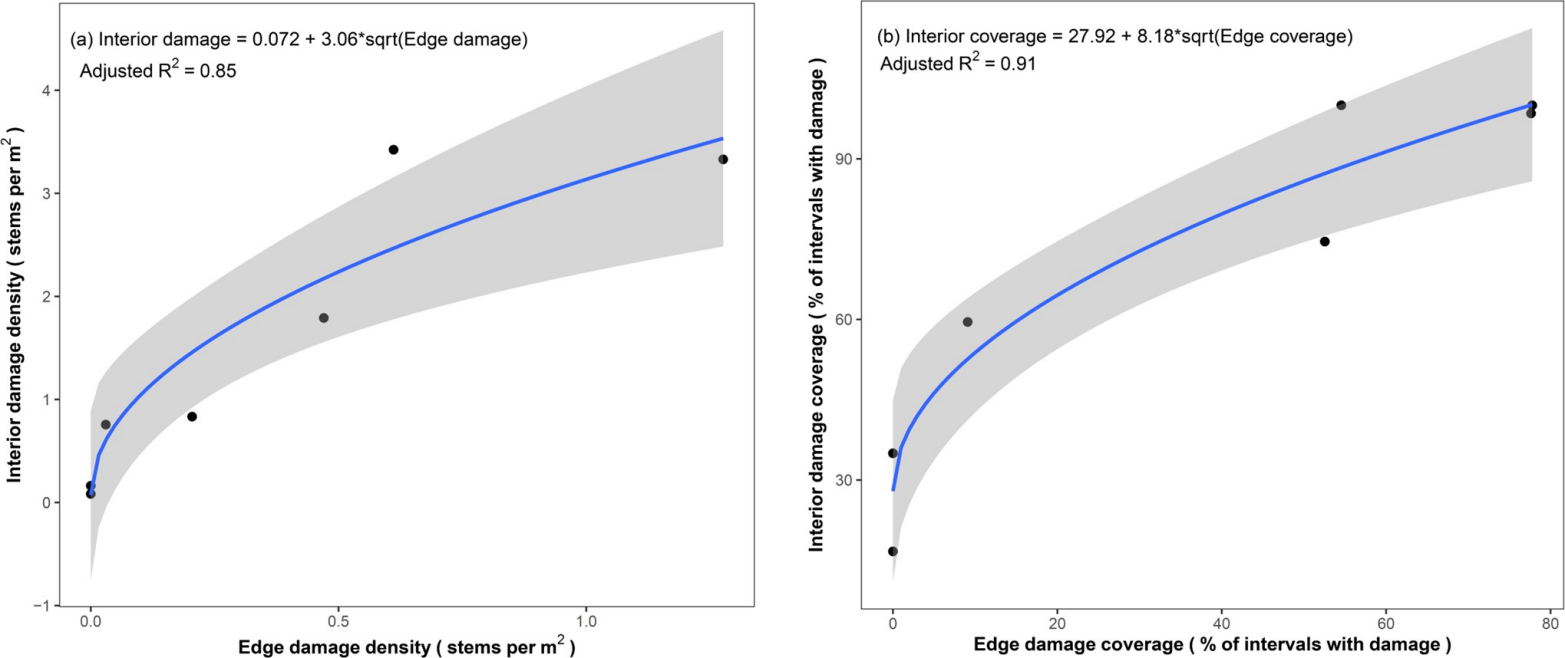
Relationship of feeding damage and damage coverage caused by released biological control agents between patch edges and interior areas of *Phragmites* plots. Scatterplots with regression lines of edge and interior (A) stem-damage density (number of damaged stems per m^2^ of monitoring transect) and (B) stem damage coverage (% of 4 × 1 m monitoring transects with ≥ 1 damaged stems) at seven biological control release sites for introduced *Phragmites*. The shaded area depicts a 95% confidence interval.

At the seven biological control sites assessed in summer 2023 (Table 2), interior transects had higher rates of biological control agent feeding damage (t = 2.90, df = 6, p = 0.03) and damage coverage (t = 6.03, df = 6, p = 0.001) compared to edge transects. Interior damage was 4-fold higher than edge damage and interior coverage 30% higher than edge coverage (Fig 5). Edge damage was also a significant predictor of interior damage (F_1,5_ = 34.16, p = 0.002, adjusted R^2^ = 0.85), and edge coverage was a significant predictor of interior coverage (F_1,5_ = 59.97, p < 0.001, adjusted R^2^ = 0.91) (Fig 6).

### Effects of larval density on initial release success

**Fig 7:**
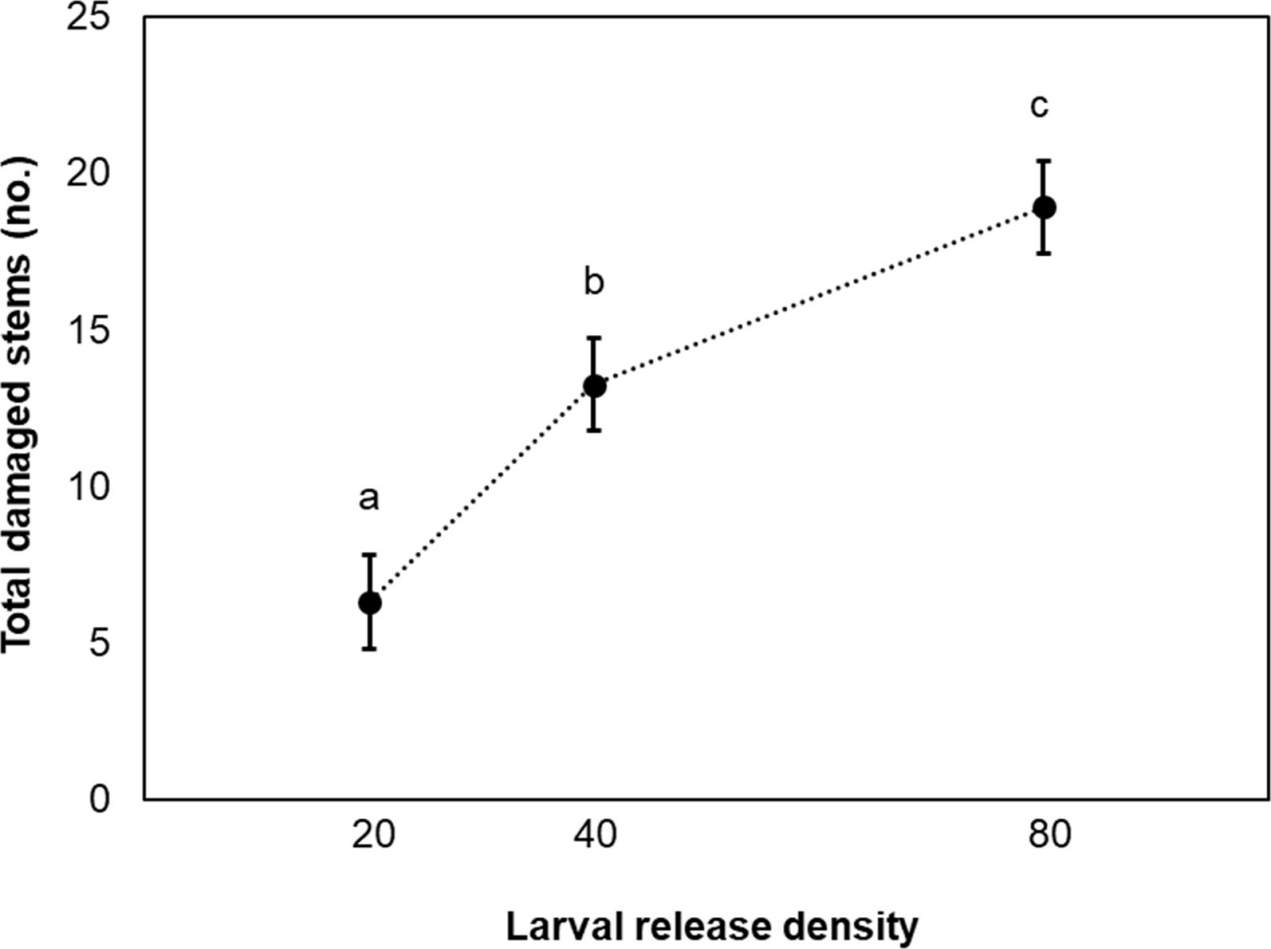
Damage of *Phragmites* stems by released biological control agents increased with higher larval release densities, albeit with diminishing returns. Total damaged stems around *Archanara neurica* release plots (*i*.*e*., the sum of damaged stems found in a 0.65-m diameter centered on the release points and during a 2-min timed search around the plots) receiving different densities of released larvae (20, 40, 80) (n = 22 plots per release density). Dots depict estimated marginal means from a mixed effects model of larval density, living stem density, living stem height, and site, error bars denote standard error, and letters indicate *post-hoc* groupings (means that do not share a letter are statistically significantly different).

Larval release density affected the number of damaged stems around release points (F_2,55.44_ = 25.77, p < 0.001), as did the density of living introduced *Phragmites* stems at the time of release (F_1,33.20_, p = 0.004, β = 0.11, SE = 0.04). Based on the estimated marginal means of the model, damage increased with the number of larvae released. However, the amount of damage increased 2.1-fold when doubling the release density from 20 to 40 larvae but only increased by 1.4-fold when doubling again from 40 to 80 larvae (Fig 7). Independent of larval release density, the total number of damaged stems increased moderately with the density of living introduced *Phragmites* stems (r_s_(64) = 0.39, p = 0.001). Mean stem height at the time of release was 50 ± 14 cm and was not a significant predictor of feeding damage (F_1,32.74_ = 2.92, p = 0.10).

## Discussion

### Initial monitoring demonstrates release and overwintering success

Long-term monitoring is essential to evaluate the success of biological control programs and invasive weed management [19, 44]. Immediate post-release and subsequent annual monitoring to assess agent presence and activity have therefore been a priority for the program targeting introduced *Phragmites* in Ontario, Canada. Initial release-point monitoring of 25 sites where *A*. *neurica* and *L*. *geminipuncta* were released revealed detectable *Phragmites* damage in 92% of the sites during the year of agent release. Those sites without detectable release-point damage experienced unplanned site disturbances or did not use the currently recommended egg and larval release techniques [36]. At all sites where damage was observed in the year of release, agent activity was also observed in subsequent years, with the density of damage often increasing between years two and three. The amount of damage caused by agent feeding observed immediately post-release was strongly predictive of the amount of damage that would be seen in the subsequent year of monitoring, which in turn was predictive of the following year. The combination of dedicated release point and patch-scale monitoring protocols was effective and efficient at assessing agent feeding damage across a growing number of large weed populations. This practical and scalable approach could be adapted to monitor the progress of other weed and biological control systems [44].

These initial results are highly encouraging for the *Phragmites* biological control program. First, they underline that successful release methods have been developed for *A*. *neurica* and *L*. *geminipuncta* [36]. Second, they confirm successful reproduction and overwintering of both *A*. *neurica* and *L*. *geminipuncta* in their introduced range, suggesting broad climatic compatibility [45] and phenological synchrony across southern Ontario [46, 47]. Finally, our results indicate that releases of even relatively small numbers of agents are sufficient to initiate a population, and that achieving significant damage in the year of release also tends to yield a proportionate increase in damage the following year. The predictive capacity of initial monitoring results will be useful to decide whether a site with low initial damage post-release may benefit from supplementary releases the following year to improve chances of agent establishment.

### Patterns of feeding damage to inform monitoring and agent recollection

Release sites with multiple years of monitoring data reveal several patterns of within-site agent feeding damage that can improve monitoring and harvesting of agents from nurse sites. Monitoring results from the second and third years after release suggest that the biological control agents are highly mobile, with feeding damage found in 100% of monitoring intervals at several sites as early as two- or three-years post-release. While there was a positive correlation between stem feeding damage density and damage coverage, even sites with low densities of feeding demonstrated relatively high dispersal throughout sites. Additionally, although the majority of agents were released within 1 to 5 m of patch edges, damage density and coverage were generally higher in patch interiors, indicating the efficient spread of agents throughout *Phragmites* infestations.

These findings also suggest larval or oviposition preferences for microsites in the interior of patches, or differential larval/egg survival associated with interior locations. These differences may be due to within patch variation in factors such as stem density, stem diameter, predation, or wind disturbance. A priority for future research will be to investigate within-site agent habitat selection, survival, and dispersal. Because most initial larval feeding damage is observed within ~ 3 m of the release points (McTavish, unpublished data), adult flight may be the principal contributor to within patch dispersal of *A*. *neurica* and *L*. *geminipuncta*. While adult flight is currently expected to be the primary means of dispersal of *A*. *neurica* and *L*. *geminipuncta*, many stem-boring lepidopteran larvae can disperse relatively long distances by crawling or “ballooning” [48–50]. Identifying the primary mode of within and between patch spread will contribute to understanding population growth patterns that will become evident during longer-term impact assessments.

These initial insights into the within-site patterns of agent feeding damage will greatly enhance the introduced *Phragmites* biological control program. First, damage density and coverage can be monitored at the edges of less accessible sites as a potential metric of overall site condition. Second, these results will help identify where to best collect agents for redistribution from potential biocontrol nurse sites to help grow the program to a landscape scale [51, 52]. Until now, all releases of *A*. *neurica* and *L*. *geminipuncta* in Canada have been insects reared in laboratories either at CABI, Switzerland or the University of Toronto. Understanding within site habitat selection and dispersal could help guide harvesting of field-adapted agent eggs from the field, reducing rearing costs and potentially preventing declines in agent fitness associated with long-term captive rearing [53–55].

### Intermediate larval densities as an optimal release strategy

Given that we found larvae to be the leading release strategy for *A*. *neurica* and *L*. *geminipuncta* [36], in 2023 we used *A*. *neurica* to refine an optimal density for releasing larvae. While releasing more larvae consistently produced more feeding damage immediately post-release, intermediate release densities proved to be the most efficient use of larvae. Our results indicated that a single release of 40 *A*. *neurica* larvae produced slightly more damage than two releases of 20 larvae. However, a single release of 80 larvae did not generate as much damage as two releases of 40 larvae. Based on these results, releases of 40 larvae appear to be optimal, providing sufficient insects to presumably survive various sources of mortality such as predation [25, 29, 32] while limiting losses to suspected intraspecific competition [56].

Results of our larval density release experiment also suggested that greater damage will occur when more introduced *Phragmites* stems are available in a 0.65-m diameter quadrat centered on the release point. Given that stem phenology during the early season can influence release success [36], these findings further demonstrate the importance of releasing an appropriate density of insects into a microsite with a sufficient number of host stems at appropriate maturity. Surprisingly, the stem height of introduced *Phragmites* at the time of agent release, however, was not a significant predictor of post-release feeding damage. This suggests that the releases were successfully conducted at times when there was a sufficient density of phenologically appropriate introduced *Phragmites* stems, or that the agents can be released into patches with a broader range of stem heights than expected with good results. Because of its strict solitary feeding and cannibalism, we speculate that the performance of *A*. *neurica* may be more constrained by intraspecific competition for resources than that of *L*. *geminipuncta* [56], and therefore it will be more sensitive to higher release densities. We therefore recommend that future experiments investigate the sensitivity of both species to phenological variation in introduced *Phragmites* stem density and height at the time of release.

### Future research and recommendations

Our results document an extremely encouraging start to the *Phragmites* biological control program in Canada, with both *A*. *neurica* and *L*. *geminipuncta* inflicting detectable feeding damage on introduced *Phragmites* that persists through the first three years of monitoring post-release. Annual monitoring of agent feeding activity will be continued at all sites to evaluate whether agents continue to persist and increase in activity at release locations. With agents now persisting across release sites for multiple years, additional standardized monitoring protocols are being developed and implemented to evaluate long-term agent demographics, agent dispersal, impacts on introduced *Phragmites* populations, and plant community responses to biological control. Going forward, another key area of study will be the ability of the two agents to establish and thrive across the wide range of conditions in which introduced *Phragmites* grows in North America. This will include study of the agents’ response to variation in introduced *Phragmites* densities, stem sizes, and hydrological conditions, as well as the potential to integrate biocontrol with other management tools. While most of the releases in Canada to date have been a single species, future releases will also investigate the potential interactive impacts and performance of releasing one or both species together under a range of environmental conditions.

Finally, there are recommendations for the ongoing expansion of the *Phragmites* biological control program based on the early years of monitoring. First, the data support a combination of large and small releases of *A*. *neurica* and *L*. *geminipuncta*. Large releases will help achieve the high initial damage and growth required to generate robust “nurse sites” for further agent redistribution, while small releases are expected to be sufficient to establish a broader network of inoculated sites across the landscape. Second, we recommend that *A*. *neurica* larval releases use iterations of 40 larvae per release point as this was an effective and efficient release density. Finally, efforts to collect eggs from “nurse sites” should focus on stems from patch interiors, where agent activity is highest. Transferring agents between weed infestations has been used to great effect in past successful weed biological control programs including purple loosestrife [57], leafy spurge [58], and knapweed [59]. Our study has demonstrated that we can get the *Phragmites* biocontrol agent insects to persist on the landscape. Developing efficient methods to locate and recollect the insects from nurse sites is the next key step for biological control of introduced *Phragmites* in North America.

## Supporting information

S1 Table Site permit summary for *Archanara neurica* and *Lenisa geminipuncta* biological control releases from 2019 to 2023 in Ontario, Canada. (DOCX)

S2 Table Arrangement of transects and monitoring intervals for patch-level monitoring of *Archanara neurica* and *Lenisa geminipuncta* biological control release sites from 2019 to 2023 in Ontario, Canada. (DOCX)
