# Supplementary material for: Current status of biological control of introduced Phragmites in Canada: Insights from initial years of post-release monitoring and a larval density release experiment
Source: PLoS One. 2024 Dec 18;19(12):e0315071. doi: 10.1371/journal.pone.0315071 (PMC11654973; doi:10.1371/journal.pone.0315071)
Supplement: S1 Table — (DOCX) [file pone.0315071.s001.docx]

**S1 Table. Site permit summary for *Archanara neurica* and *Lenisa geminipuncta* biological**

**control releases from 2019 to 2023 in Ontario, Canada.**

| **Site** | **Permit received** | **Permitting authority** |
| --- | --- | --- |
| P01: Davern | Not required | Private property |
| P02: Aurora | Not required | Ducks Unlimited Canada |
| P03: Wainfleet | Not required | Public roadside |
| P04: Sinclair Campbell | Not required | Ducks Unlimited Canada |
| P05: Oshawa | Not required | Ducks Unlimited Canada |
| P06: Koffler | Yes | Koffler Scientific Reserve |
| P07: Aultsville | Not required | Ducks Unlimited Canada |
| P08: Madoc | Not required | Public roadside |
| P09: Scarborough | Yes | University of Toronto Scarborough |
| P10: Zoo | Not required | Toronto Zoo |
| P11: Waterloo | Not required | University of Waterloo |
| P12: rare | Yes | rare Charitable Research Reserve |
| P13: Dunnville | Not required | Ducks Unlimited Canada |
| P14: Cranberry | Yes | Central Lake Ontario Conservation Authority |
| P15: Mac Coutts | Yes | Grand River Conservation Authority |
| P16: Collavino | Yes | Essex Region Conservation Authority |
| P17: Cooper | Not required | Ducks Unlimited Canada |
| P18: Whitby | Yes | Town of Whitby |
| P19: Brickworks | Yes | City of Toronto |
| P20: St. Lukes | Not required | Ducks Unlimited Canada |
| P21: Brimblecombe | Not required | City of Guelph |
| P22: North Bay | Yes | North Bay-Mattawa Conservation Authority |
| P23: Garrard | Not required | Public roadside |
| P24: Nichol | Not required | Public roadside |
| P25: Victoria | Not required | Public roadside |
| P26: Gordon | Not required | Public roadside |
| P27: Lakeridge | Not required | Public roadside |
| P28: Cochrane | Not required | Public roadside |
| P29: Brooklin | Not required | Public roadside |
| P30: Donkey | Not required | Ducks Unlimited Canada |
