# Supplementary material for: Current status of biological control of introduced Phragmites in Canada: Insights from initial years of post-release monitoring and a larval density release experiment
Source: PLoS One. 2024 Dec 18;19(12):e0315071. doi: 10.1371/journal.pone.0315071 (PMC11654973; doi:10.1371/journal.pone.0315071)
Supplement: S2 Table — (DOCX) [file pone.0315071.s002.docx]

**S2 Table. Arrangement of transects and monitoring intervals for patch-level monitoring of *Archanara neurica* and *Lenisa geminipuncta* biological control release sites from 2019 to 2023 in Ontario, Canada.**

| **Site** | **Main transect arrangement**  **(year: number of monitoring intervals)** | **Additional perimeter transects for interior-edge comparison**  **(year: number of monitoring intervals)** |
| --- | --- | --- |
| P01: Davern | 1 combined interior/edge (2022: 14) | N/A |
| P02: Aurora | 4 interior (2022: 38, 2023: 39) | 1 edge (2023: 18) |
| P03: Wainfleet | 1 interior (2022, 2023: 18) | 1 edge (2023: 23) |
| P04: Sinclair Campbell | 2 interior (2022: 20, 2023: 16) | N/A |
| P05: Oshawa | 2 interior (2022: 53, 2023: 42) | 2 edge (2023: 33) |
| P07: Aultsville | 1 interior, 1 edge (2022: 15, 2023: 16) | N/A |
| P08: Madoc | 1 interior (2022: 17, 2023: 16) | N/A |
| P09: Scarborough | 4 interior (2023: 20) | 1 edge (2023: 17) |
| P10: Zoo | 1 combined interior/edge (2023: 14) | 1 edge (2023: 11) |
| P11: Waterloo | 5 interior (2023: 66) | 1 edge (2023: 76) |
| P12: rare | 6 interior (2023: 55) | 2 edge (2023: 59) |

Main transect arrangement refers to the number and location (i.e., patch interior, edge, or combined) of the transects used to collect patch-level monitoring data for sites year two onwards following the release of biological control agents. Additional perimeter transects refers to extra transects around the edge of certain patches to compare biological control agent activity between the interior and edge habitats of introduced *Phragmites* patches. The total number of 4 × 1 m monitoring intervals placed on transects are given in parentheses for each year; the total number of monitoring intervals can vary slightly year-to-year depending on the exact placement of the transects.
